# Self-management education for adults with poorly controlled epilepsy (SMILE (UK)): statistical, economic and qualitative analysis plan for a randomised controlled trial

**DOI:** 10.1186/s13063-015-0788-9

**Published:** 2015-06-12

**Authors:** Nicholas Magill, Leone Ridsdale, Laura H. Goldstein, Paul McCrone, Myfanwy Morgan, Adam J. Noble, Gus Baker, Mark Richardson, Stephanie Taylor, Sabine Landau

**Affiliations:** Department of Biostatistics, Institute of Psychiatry, Psychology and Neuroscience, King’s College London, PO 20, , Denmark Hill Campus, 16 De Crespigny Park, London, SE5 8AF UK; Department of Basic and Clinical Neurosciences, Institute of Psychiatry, Psychology and Neuroscience, King’s College London, PO 43, , Denmark Hill Campus, 16 De Crespigny Park, London, SE5 8AF UK; Department of Psychology, Institute of Psychiatry, Psychology and Neuroscience, King’s College London, PO 77, , Denmark Hill Campus, 16 De Crespigny Park, London, SE5 8AF UK; Department of Health Service and Population Research, Institute of Psychiatry, Psychology and Neuroscience, King’s College London, PO 24, , Denmark Hill Campus, 16 De Crespigny Park, London, SE5 8AF UK; Division of Health and Social Care Research, School of Medicine, King’s College London, 7th Floor Capital House, 42 Weston Street, London, SE1 3QD UK; Department of Psychological Sciences, Institute of Psychology, Health and Society, The Whelan Building, University of Liverpool, Brownlow Hill, Liverpool, L69 3GB UK; Department of Molecular and Clinical Pharmacology, University of Liverpool, Ashton Street, Liverpool, L69 3GE UK; Barts & The London School of Medicine and Dentistry, Centre for Primary Care and Public Health, Blizard Institute, Abernethy Building, 2 Newark Street, London, E1 2AT UK

**Keywords:** Statistical analysis plan, SMILE trial, epilepsy, self-management education, randomised controlled trial

## Abstract

**Background:**

There is a need to test the effectiveness of new educational interventions for people with poorly controlled epilepsy. The SMILE (self-management education for adults with poorly controlled epilepsy) trial evaluates a complex service intervention that involves a 2-day self-management course with the aim of improving quality of life and clinical outcomes. This article describes the statistical, economic, and qualitative analysis plan for the trial.

**Methods and design:**

SMILE is a pragmatic, parallel design, two-arm, multi-centre randomised controlled superiority trial of a group-based interactive course compared with treatment as usual for people who have experienced two or more seizures in the past 12 months.

**Results:**

A summary of the objectives and design of the trial are reported as well as the manner in which the data will be summarised and inferentially analysed. This includes the type of modelling that will be employed for each of the primary and secondary outcomes and the methods by which the assumptions of these models will be checked. Strategies are described for handling clustering of outcome data, missing observations, and treatment non-compliance.

**Conclusion:**

This update to the previously published trial protocol provides a description of the trial analysis which is transparent and specified before any outcome data are available. It also provides guidance to those planning the analysis of similar trials.

**Trial registration:**

Current Controlled Trials ISRCTN57937389; date assigned: 27 March 2013.

## Update

### Background

#### Epilepsy

Epilepsy is a long-term neurological condition, which affects approximately 1 % of the UK population [1]. After diagnosis, roughly 40 % of patients continue to experience at least two seizures per year [2] and the consequences for this group include elevated risks of injury, depression, and premature death [1]. From the perspective of health service provision, poorly controlled epilepsy is expensive (€2,000–€11,500 per case in 2004) [3]. The overwhelming majority of admissions for epilepsy are on an emergency basis [4], making it the sixth most common cause of emergency admission for chronic conditions in the UK [5] and adding considerably to health-care cost.

Providing patients with the ability to manage long-term conditions and reducing emergency admissions are key aims of the National Health Service (NHS). For people with poorly controlled epilepsy, this means increasing patients’ confidence in their ability to recognise triggers for seizures [6] and improving their adherence to antiepileptic drugs (AEDs) [7, 8]. Routine NHS policy for another chronic illness, diabetes, is the offer of group education (e.g., diabetes education and self-management for ongoing and newly diagnosed, or DESMOND [9]), and there is now demand for similar courses for those with epilepsy. This is supported by the finding that a third of those with poorly controlled epilepsy have been told little about the disease and the side effects of AEDs [10].

Of a number of epilepsy interventions reviewed by the Cochrane collaboration [11, 12], only one has been thoroughly assessed and shown potential benefit in the UK. This group intervention was developed in Germany, where it was called Modular Service Package in Epilepsy (MOSES) [13]. A trial of this reported improved knowledge about the condition, enhanced control of seizures, and better acceptance of AEDs. The intervention has been adapted for use in the UK and is referred to here as the SMILE (self-management education for adults with poorly controlled epilepsy) trial.

### SMILE trial

The SMILE trial is a parallel design, two-arm, multi-centre randomised controlled superiority trial of a 2-day self-management course for adults with poorly controlled epilepsy. Participants are randomly allocated to receive either SMILE and treatment as usual (TAU) or TAU alone, and randomisation is stratified by treatment centre. The aim is to test whether the intervention affects quality of life, clinical outcomes, and cost-effectiveness of health service use at 6 and 12 months after randomisation. Blinding is planned for outcome assessors and the trial statistician. See protocol article for further details [14].

This article describes the statistical analysis plan for the main analyses of the trial. The plan was finalised and approved by the Trial Steering Committee on 3 March 2014, before any outcome data were available.

### Research questions

#### Primary objective

The primary objective is to examine the treatment difference in Quality of Life in Epilepsy-31 (QOLIE-31) [15] scores between participants allocated to self-management education (SMILE) versus those allocated to TAU at 12 months after randomisation.

#### Secondary objectives

The secondary objectives are the following:

1. To investigate the treatment difference in QOLIE-31 between participants allocated to SMILE and those allocated to TAU at 6 months after randomisation.
2. To investigate the treatment effect on seizure frequency as recorded using the scale of Baker et al. [16] between participants allocated to SMILE and those allocated to TAU at 6 and 12 months after randomisation.
3. To investigate the treatment effect on seizure frequency as recorded using the scale of Thapar et al. [17] between participants allocated to SMILE and those allocated to TAU at 6 and 12 months after randomisation.
4. To investigate the treatment effect on time elapsed since last seizure of allocation to SMILE compared with those offered TAU at 6 and 12 months after randomisation.
5. To investigate the treatment difference in self-perceived impact of epilepsy (as measured by the Impact of Epilepsy scale [18]) between those allocated to SMILE and those offered TAU at 6 and 12 months after randomisation.
6. To investigate the treatment difference in medication management as measured by the Epilepsy Self-Management Questionnaire [7] between participants allocated to SMILE and those allocated to TAU at 12 months after randomisation.
7. To investigate the treatment difference in medication adverse events (AEs) as measured by two questions from QOLIE-31 [15] between participants allocated to SMILE and those allocated to TAU at 6 and 12 months after randomisation.
8. To investigate the treatment effect on psychological distress as measured by the Hospital Anxiety and Depression Scale (HADS) [19] between participants allocated to SMILE and those allotted to TAU at 12 months after randomisation.
9. To investigate the treatment difference in perceived stigma as measured by Jacoby’s Stigma of Epilepsy scale [20] between participants allocated to SMILE and those allotted to TAU at 12 months after randomisation.
10. To investigate the treatment effect on self-efficacy in management of epilepsy (using the mastery/control of epilepsy scale [21]) between participants allocated to SMILE and those allocated to TAU at 12 months after randomisation.
11. To investigate the treatment difference in cost-effectiveness of health service use between participants randomly assigned to SMILE and those randomly assigned to TAU at 12 months after randomisation.
12. To conduct an in-depth qualitative interview study to investigate SMILE users’ views on their participation in, and the perceived benefits of, the intervention.

See protocol for further details of measures [14].

## Trial design

### Recruitment procedure and randomisation

**Fig. 1:**
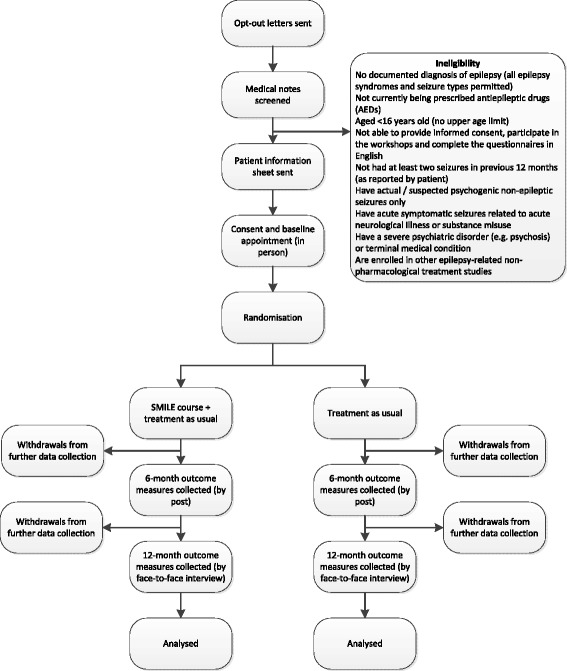
CONSORT (Consolidated Standards of Reporting Trials) diagram. *SMILE* self-management education for adults with poorly controlled epilepsy

Research workers visit participants at their home or a place of their choice and explain the trial in detail. Those individuals who meet the eligibility criteria are asked to provide informed written consent and baseline assessments. Consenting individuals who provide baseline data are randomly assigned to one of the treatment arms in a 1:1 ratio. See Fig. 1 for an illustration of the process by which patients are screened, recruited, randomly assigned, and followed up. Randomisation is at the patient level and is performed by using an online randomisation system set up by the King’s Clinical Trials Unit (KCTU) at the Institute of Psychiatry, Psychology and Neuroscience in London. Randomisation is stratified by treatment centre. A set of patients is randomly assigned with fixed block sizes of two to ensure that equal numbers of patients are allocated to the two arms within each treatment centre. The procedure is as follows: on receipt of the baseline questionnaire and after between 14 and 24 participants have been recruited to a site, the trial coordinator electronically submits details of the set of participants to the KCTU. The system immediately notifies the trial coordinator of successful randomisation and the trial arm to which the participant has been allocated. A record of the allocation outcome is kept in the KCTU randomisation database and later linked to the main dataset.

Ethical approval was given by the National Research Ethics Service London – Fulham (reference 12/LO/1962).

### Sample size

The primary intention-to-treat (ITT) analyses will compare two equal-sized treatment arms, treatment or control, on the quality-of-life scale (QOLIE-31) at 12 months. Two drug trials that used this scale as an outcome showed standardised effect sizes of d = 0.33 [22] and 0.59 [23]. An overall sample size of 320 participants (randomly assigned 1:1) would provide 91.3 % power to detect an effect size of d = 0.40 on the QOLIE-31 using an analysis of covariance with two-sided 5 % significance tests. A value of d = 0.40 corresponds to a change of around 6–7 points on the overall quality-of-life score.

Since the active treatment is a group treatment delivered by different educational facilitators within sites, we must allow for standard error inflation due to training group effects. Assuming an average group size of 10 people with epilepsy and an intra-group correlation between QOLIE-31 scores of intra-class correlation coefficient (ICC) equal to 0.025 (a correlation of 0.05 would be expected for intermediate outcomes but would be lower for distal outcomes such as quality of life [24]), we estimated that 160 patients in the control arm and 16 groups of 10 patients in the SMILE arm would provide 91.3 % power to detect an effect of d = 0.40.

The estimated attrition rate was based on data from a study of rehabilitation for people with severe epilepsy which had a 25 % loss at 1 year (NIHR SDO 08/1815/234). Therefore, to ensure adequate and equal-sized groups, a sample of 428 patients is required (*n* = 320/0.75; 214 patients per arm).

### Timing and visit windows of follow-up

Participants will complete a limited selection of follow-up measures at 6 months after randomisation by using a questionnaire sent to them in the post. This comprises quality of life, seizure frequency, time elapsed since last seizure, and impact of epilepsy. The full range of follow-up measures will be completed at 12 months after randomisation by face-to-face interview with a research assistant. The aim is for follow-up assessments to be collected within 3 weeks of the intended date.

### Minimising attrition

It is expected that some participants will be lost to follow-up and this has been accounted for in the sample size calculation. However, every effort will be made to minimise such attrition, by following the recommendations of recent research [25]. A sequence of telephone reminders will be used to maximise the collection of follow-up data, especially at the 6-month assessment. This assessment has been specifically condensed in order to reduce patient burden. An additional measure to increase adherence is the distribution of £20 vouchers following the 12-month assessment.

### Blinding

Outcome assessors and the trial statistician will be blind to participants’ allocated treatment arm. At the start of each follow-up assessment, interview participants are requested not to reveal to the researcher which group they were allocated to. If a researcher is unblinded at the 6-month outcome visit, a different researcher will conduct the next follow-up assessment. Additionally, the trial databases have been designed specifically to enable data entry without unblinding researchers. This has been achieved by creating separate databases for recording outcome data, course attendance, and educational facilitator information. We are assessing how well researchers were kept blind by asking the assessor to make a guess as to which treatment each participant was allocated to at the end of follow-up.

### Reporting

The trial results will be reported in a manner that is consistent with the recommendations of the updated CONSORT (Consolidated Standards of Reporting Trials) guidelines for parallel group trials [26]. Additionally, the report will follow the CONSORT extensions on pragmatic and non-pharmacologic trials [27, 28].

### Description of data

#### Baseline comparability of randomly assigned groups

Baseline descriptions of participants’ demographic and clinical data will be reported by treatment group and overall. Minima and maxima, means and standard deviations, and medians and quartiles will be used for continuous variables as appropriate. Frequencies and proportions will be presented for categorical variables. No significance testing will be used to test baseline differences between the trial arms.

#### Adherence to allocated treatment

Adherence is defined as being in attendance at the beginning and end of each of the two days of the self-management course. Adherence versus non-adherence with the treatment will be described in terms of baseline variables. The reasons for withdrawal from treatment will be summarised. Treatment adherence will be described by using attendance and reasons for non-attendance.

#### Loss to follow-up and missing data

Withdrawal from trial follow-up (attrition) will be reported by intervention group. Moreover, the proportions of participants who are missing values on each variable will be summarised by trial arm and time point.

The baseline characteristics of those missing follow-up will be compared with those with complete follow-up. The relationship between baseline characteristics and missing data will also be investigated graphically. Factors affecting missingness will be examined by using logistic regression for a missingness variable. This will be done by generating a binary variable for missingness at 12 months after randomisation and regressing this on pre-randomisation (baseline) variables. All outcome variables will be assessed before choosing a representative selection.

The relationship between compliance and loss to follow-up will be assessed. This will be done by using binary variables for completion of all training sessions and for drop-out at 12 months. The relationship between these variables will be tested by using a chi-squared test. The results of these analyses will inform the need to use multiple imputation (MI) in the formal analysis. This is because these post-randomisation variables cannot be included as covariates in the model without changing the meaning of the results.

#### Adverse events

AEs, adverse reactions, serious AEs, and serious adverse reactions will be summarised by treatment arm.

#### Descriptive statistics for outcome measures

Each of the outcome measures will be described by treatment group and time point. Means and standard deviations or medians and interquartile ranges will be used for continuous variables; box plots, histograms, and Q-Q plots will be used to assess whether the distribution of a variable is normal. Frequencies and proportions will be used to describe categorical variables.

### Inference and modelling

#### Inferential analysis

The main statistical analyses will estimate the difference in mean outcomes between patients randomly assigned to SMILE and TAU versus TAU alone by ITT (i.e., treatment groups defined by result of randomisation rather than treatment received) at the various post-treatment observation time points. This will be done by using mixed-effects linear regression modelling for longitudinal data, including outcome measures at all time points and conditioning on the stratification variable (treatment centre) and baseline measures. Group difference estimates and associated confidence intervals will be reported.

Missing post-randomisation assessments will be dealt with by fitting adequate linear mixed models to all the variables by using maximum likelihood (ML) methods. Such an approach provides valid inferences under the assumption that the missing data mechanism is ignorable (missing at random, or MAR) and provided that predictors of missingness are included as covariates in the model.

Group difference estimates and associated 95 % confidence intervals will be reported. The trial statistician will remain blind until the main analyses have been completed. Some analyses, such as modelling training group effects, estimating complier average causal effects (CACEs), and summarising numbers trained by each educational facilitator, cannot be performed blind. Such analyses will be carried out last in order to preserve blindness for as long as possible.

The significance level will be 5 % (two-sided) for the primary outcome. Secondary analyses will be carried out at the 5 % level but will have to be interpreted with care as multiple testing is not taken account of. Sensitivity analyses will be used to assess the robustness of conclusions to non-ignorable missing outcome data.

#### Analysis of primary outcome

The analysis population will include all randomly assigned patients. The primary outcome is quality of life, as measured by the QOLIE-31, at 12 months after randomisation. Quality of life at both post-treatment time points (6 and 12 months after randomisation) will be modelled simultaneously. These outcomes will constitute the dependent variable, and quality of life at baseline, treatment centre, baseline predictors of drop-out, trial arm, time dummy variables, and a treatment*time interaction term will be included as explanatory variables. This last term will allow the model to provide the treatment effect estimate at the primary time point (12 months after randomisation). The covariance matrix of the repeated measures will be carefully modelled: an unstructured covariance matrix and the covariance matrix implied by a participant-varying random intercept model will be formally compared, and the best covariance structure will be identified. This analysis is valid provided that missing values in quality-of-life outcomes are MAR. This is to say that, given the observed variables that have been included in the analysis model, the missingness pattern does not depend on any unobserved/unmodelled data.

The relationship between baseline variables and missing outcome data will be assessed by using logistic regression with an outcome variable that represents whether outcome quality-of-life data are present or missing. Should any baseline variables be predictive of missingness, these will be included in the model as covariates. Should the post-treatment variable “compliance with treatment” predict missingness, MI will be used to approximate an ML estimator under this form of MAR [29]. The impact of departures from MAR on treatment effects will be assessed by using sensitivity analysis [30].

Potential clustering due to participants attending the same educational group (and therefore sharing the same interventionist) in the SMILE arm will also be assessed by exploring the variability between these groups. This will be summarised by using the ICC. It is expected that a statistical dependence will exist and that this will need to be addressed in the modelling process. Therefore, it is anticipated that models will need to include random effects that vary with educational group in this trial arm.

#### Analysis of secondary outcomes

Secondary patient outcomes relating to the quality of life (at 6 months after randomisation), impact of epilepsy, medication management, psychological distress, and mastery/control of epilepsy will be analysed by using linear mixed models and an approach similar to that described above.

Medication adverse effects and perceived stigma are unlikely to be normally distributed. Transformations will be investigated for these outcomes. Failing this, other types of mixed models, such as a mixed logistic model, will be considered.

Seizure frequency as measured by using the scale of Thapar et al. is a count variable and will be analysed by using a type of Poisson mixed model, and allowance will be made for overdispersion/varying exposure periods. Seizure frequency using the scale of Baker et al. is an ordinal variable and will be analysed by using a mixed ordinal logit model.

Time (days) elapsed since last seizure is measured pre-randomisation (at baseline) and at 6 and 12 months after randomisation. Such a variable will be analysed after a log transformation. If the data are truncated, survival modelling will be considered.

### Statistical considerations

#### Stratification and clustering

Randomisation is stratified by treatment centre (of which there are up to 15). Therefore, it is important to include this variable as a covariate in the modelling process. The structure of the majority of the data is longitudinal, and repeated measurements are taken at baseline and 6 and 12 months after randomisation. This correlation between participants’ repeated observations is being taken into account by a modelling process for the covariance matrix. It is also possible that clustering will be seen within educational facilitators/educational groups within the SMILE arm.

#### Missing scale items

The number (percentage) with complete data will be reported. The ideal approach is to use missing value guidance provided by the developers of the scales. When such guidance is not available, scales will be prorated for an individual if not more than 20 % of items are missing. For example, in a scale with 10 items, prorating will be applied to individuals with one or two items missing. The average value for the eight or nine complete items will be calculated for that individual and used to replace the missing values. The scale score will be calculated on the basis of the complete values and these replacements.

#### Missing baseline data

Missing baseline data should not be a problem. However, if we encounter missing baseline values of outcome variables, these can be singly imputed according to White and Thompson [31] without incurring bias in trial arm effect estimates.

#### Missing outcome data

Where there are two outcome time points, missing post-randomisation assessments will be dealt with by fitting linear mixed models to all the available data by using maximum likelihood methods. Such an approach provides valid inferences under the assumptions that the missing data mechanism is ignorable (or MAR) and that pre-randomisation data are available for the scale. If post-treatment variables such as compliance with treatment are found to be predictive of drop-out, multiple imputation will be considered. This will be assessed through the use of a chi-squared test of independence.

#### Method for handling multiple comparisons

For the primary outcome, no formal adjustment of the *P* value for multiple testing is necessary. The analyses of the secondary outcomes will also not be adjusted for multiple outcome comparisons. Thus, care should be taken in the interpretation of inferences for the numerous secondary outcomes. (Results will need to be interpreted as hypotheses-generating and subject to replication.)

#### Method for handling non-compliance

In addition to the primary ITT analysis, the effect of actually receiving treatment as defined in the protocol (its efficacy) will be estimated for the primary outcome. If non-adherence with the active treatment is high, a CACE-type analysis will be considered. Specifically, instrumental variable (IV) methods will be used to assess the efficacy of the SMILE treatment. Such methods evaluate the causal effect of SMILE on a clinical outcome in the subpopulation who would comply with SMILE. The application of IV methods for explanatory evaluation of randomised controlled trials has been advocated because random allocation itself provides a strong instrument for treatment receipt [32]. IV regression will be carried out by using the two-stage least squares estimator as implemented in the Stata command “ivregress 2sls”.

#### Model assumption checks

The models assume normally distributed outcomes; this will have been checked when describing the data, and if substantial departures from normality occur, transformations will be considered. Residuals will be plotted to check for normality and inspected for outliers. This is expected to be unnecessary for the primary outcome but will be undertaken partly in order to explore the data and to maintain a consistent method for all outcomes.

### Sensitivity analyses

The impact of departures from MAR on treatment effects of continuous outcomes will be assessed by using sensitivity analysis. This will be done for the quality-of-life outcomes and will be based on the investigators’ opinion about the range of possible mean differences in outcome between those with missing data and those with observed values in the two trial arms [30].

### Planned subgroup analyses

The trial has not been designed (powered) to assess treatment effects within subgroups. As a consequence, no such subgroup analyses are described.

### Software

Data management will employ an online data collection system for clinical trials (MACRO; InferMed Limited, London, UK). This is hosted on a dedicated server at King’s College London and managed by the KCTU. The KCTU Data Manager will extract data periodically as needed. Stata will be used for data description and the main inferential analysis.

### Economic analysis plan

#### Heath economic objectives (secondary objective 11)

We will take both a health service and a societal perspective in the economic evaluation. The costs of the intervention will be calculated by taking into account staff time needed for training, supervision, and delivery of the educational session and will also include overheads and capital costs. The cost per session will be estimated by combining the above information with activity data. The Client Service Receipt Inventory will be adapted and used to record the use of other services and also unpaid carer time and time lost from work. The service use data will be combined with relevant unit cost information [33]. Lost employment costs will be calculated by combining lost work time with average wage rates. Health-care and societal costs at follow-up will be compared between the two arms by using a regression model with baseline costs controlled for. Cost data are often skewed, and we will use bootstrapping to generate 95 % confidence intervals around the cost differences. To assess cost-effectiveness, we will combine costs with data for the primary outcome measure at 12 months. Cost utility will be assessed by combining costs with quality-adjusted life years (QALYs), which will be generated from the European Quality of Life-5 Dimensions (EQ-5D) questionnaire. If the intervention results in better outcomes and lower costs, it will be considered to be “dominant”. If it results in better outcomes and higher costs, incremental cost-effectiveness ratios will be calculated to show the extra cost incurred to achieve a one-unit improvement on the QOLIE-31 or one extra QALY (both at 12 months). Uncertainty around the estimates of cost-effectiveness and cost utility will be made by taking 1000 cost-outcome combinations at random (and with replacement) from the data by using bootstrap methods and plotted on a cost-effectiveness plane. Interpretation of the results will use cost-effectiveness acceptability curves to show the probability that the intervention is the most cost-effective option for a range of different values placed on an improvement in outcome. For QALYs, the range will be £0 to £100,000. The range for improvements on the QOLIE-31 will be chosen so that values at which the intervention or TAU has a 50 % and 70 % and 90 % likelihood of being cost-effective are identified.

### Economic measures

Client Services Receipt Inventory: This will record contacts with health-care services at baseline and over the follow-up. It includes hospital admissions, contact with primary and community care, and receipt of care from family and friends. In addition, it includes lost work time.

EQ-5D: QALYs will be calculated from the EQ-5D health state classification instrument. This covers five domains: mobility, self-care, usual activities, pain/discomfort, and anxiety/depression. For each domain, the respondent chooses one of five levels of functioning, from good to poor. The five levels for each of the five domains are used to define unique health states to which a pre-estimated “utility” value will be attached.

### Qualitative analysis plan (secondary objective 12)

#### Aim

Qualitative studies are increasingly conducted as a component of the evaluation of “complex” interventions with the aim of supplementing quantitative measures and contributing to the wider implementation of health-care interventions [34]. The present study aimed to complement the quantitative measures by providing an in-depth account of (1) participants’ perceptions of what they valued and any negative aspects of the intervention and (2) whether and in what ways participants continued to make use of the training received.

## Methods

### Study group

Pilot interviews at 12 months post-intervention have indicated that participants were often not able to recall whether they had learned particular information about epilepsy and its management from the intervention or through other sources. This supports evidence that ongoing seizures do impair the laying down of memory [35]. However, reducing the period between intervention and interviews would have possible implications for subsequent outcome measurement. Therefore, we will interview the control group participants 2–6 months after they have received the control group intervention and have completed the three phases of quantitative data collection.

### Recruitment

The study will be based on around 20–24 study participants who were randomly assigned to the SMILE (UK) control group, and the precise sample number will depend on when sampling saturation has been achieved and the condition that no new issues or explanations are elicited by further interviews [36]. Participants will be selected purposively to achieve a study group that is inclusive in terms of differences in gender, age, ethnicity, severity of seizures, and depression score.

### Interviews

These will take place at participants’ homes or a convenient public place if preferred. Interviews will be semi-structured and conducted in a conversational manner with probes, facilitation, and clarification of responses as required. The topics covered will include participants’ experiences in taking part in the courses, their perceptions of things they valued and found of particular benefit and any negative aspects as well as any factors that encouraged or hindered their participation in the courses and whether and in what ways they have continued to make use of the training. The content of the course workbook will also be discussed in relation to participants’ own needs and lifestyles.

### Analysis

The interviews will be audio-recorded (with consent) and transcribed. The analysis will proceed alongside data collection and will be based on a framework approach [37]. This is suitable for small numbers of cases and ensures that each case is fully taken into account in the analysis. This analytic approach requires identifying initial themes that are then grouped into main and sub-themes. This is applied to the raw transcript data for each interview, and a thematic “chart” is created that summarises information for each theme and allows cross-case and within-case analysis through a process of constant comparison, and particular attention is paid to deviant cases. This approach to analysis can be undertaken manually or by using new framework matrices from NVivo9 or 10. Two members of the research team will participate in data analysis to reduce bias in the identification and interpretation of themes and categories.

#### Outputs

The findings of this nested qualitative study will contribute to outcome assessment of the trial by providing explanations and a greater understanding of the quantitative assessment as well as exploring issues not covered by these assessments. The findings may also be written up as a stand-alone article that examines patient-based assessments of a self-management intervention.

## Abbreviations

AE: Adverse event
AED: Antiepileptic drug
CACE: Complier average causal effect
CONSORT: Consolidated Standards of Reporting Trials
EQ-5D: European Quality of Life-5 Dimensions
ICC: Intra-class correlation coefficient
ITT: Intention to treat
IV: Instrumental variable
KCTU: King’s Clinical Trials Unit
MAR: Missing at random
MI: Multiple imputation
ML: Maximum likelihood
NHS: National Health Service
QALY: Quality-adjusted life year
QOLIE-31: Quality of Life in Epilepsy-31 scale
SMILE: Self-Management education for adults with poorly controlled epilepsy
TAU: Treatment as usual.
